# Corn gluten meal induces enteritis and decreases intestinal immunity and antioxidant capacity in turbot (*Scophthalmus maximus*) at high supplementation levels

**DOI:** 10.1371/journal.pone.0213867

**Published:** 2019-03-13

**Authors:** Nan Bai, Min Gu, Mingjie Liu, Qian Jia, Shihui Pan, Zhiyu Zhang

**Affiliations:** Marine College, Shandong University at Weihai, Weihai, Shandong Province, PR China; University of Illinois, UNITED STATES

## Abstract

Corn gluten meal (CGM) is an important alternative protein source in aquafeed production. However, in turbot (*Scophthalmus maximus*), CGM could not be effectively utilized because of its low digestibility, the reason for which is still unclear. The purpose of the present study was to investigate and elucidate the cause for the poor utilization of CGM by turbot from the view of gut health. An 8-week feeding trial was conducted with turbot individuals (initial body weight 11.4 ± 0.2 g), which were fed with one of four isonitrogenous and isolipidic diets formulated to include 0%, 21.2%, 31.8%, and 42.6% CGM to progressively replace 0%, 33%, 50%, and 67% fish meal (FM) protein in a FM-based diet, respectively. The results showed that CGM caused dose-dependent decreases in (1) growth performance, nutrient digestibility, and feed utilization; (2) activities of brush-border membrane enzymes; (3) intestinal antioxidant indices of superoxide dismutase, catalase, glutathione peroxidase, glutathione reductase activities, and reduced glutathione level; (4) intestinal immune parameters of acid phosphatase activity, complement 3, complement 4, and IgM concentrations. Dose-dependent increases in the severity of the inflammation, with concomitant alterations on microvilli structure and increasing expression of inflammatory cytokine genes of *Il-1β*, *Il-8*, and *Tnf-α* were observed but without a change in the intracellular junctions and the epithelial permeability established by the plasma diamine oxidase activity and D-lactate level examinations. In conclusion, the present work proved that CGM negatively affected the gut health of turbot by inducing enteritis and by decreasing intestinal immunity and antioxidant capacity, which could be one of the reasons for the reduced utilization of CGM by turbot.

## Introduction

Aquafeed production is rapidly increasing with the high-speed expansion of aquaculture. Hence, the growth of aquafeed industry requires sustainable feed ingredients supply, especially of protein sources [1]. Traditionally, fish meal has been used as the primary protein source in aquafeeds due to its high protein content, excellent amino-acid profile and high nutrient digestibility [2]. However, the production of fish meal seems to have reached its maximum potential, and its level has been stabilized over the last decades [3]. The unbalance between the fish meal supply and demand has stimulated the exploration of alternative protein sources for the aquaculture [4,5], and research on fish meal substitution has been an international priority for more than two decades [6].

Corn gluten meal (CGM) is the major protein portion obtained from the wet milling process for the separation of the starch, germ, protein, and fiber components from corn [7]. Compared with other vegetable protein sources, CGM is considered a cost-effective alternative protein source for aquafeed owing to its high content of available protein (60%–70% of the dry matter), low content of fiber and anti-nutritional factors, competitive price, and steady supply [8]. In some carnivorous fish, such as cobia, Japanese seabass, and sea bream, CGM has been shown to successfully replace more than half of the fish meal protein used in the diet without any negative effect on growth performance [9–11]. However, CGM could not be effectively utilized by turbot, which is considered the most important cultured flatfish in Europe and Asia with a global production of around 70,000 t per year [12]. Regost *et al*. found that CGM can replace only 33% of the fish meal protein in the diet of turbot. The authors attributed the bad performance of fish to the low digestibility of the nutrients caused by CGM inclusion [13]. In another work to assess the apparent digestibility coefficients (ADCs) of eight protein sources in turbot, CGM had the lowest ADC values of dry matter, protein, energy, and most of the assayed amino acids [14]. Low digestibility is the key factor limiting the utilization of CGM by turbot, but its causes are still unclear.

The intestine is the major site for the acquisition of food with subsequent assimilation of vital nutrients, and its digestion and absorption functions were found in a previous studies to be significantly affected by the health status [15]. Fish intestine health status was determined by endogenous and exogenous materials, in which the feedstuffs had been extensively researched [16]. It has earlier been established that the plant protein supplemented in the diet impairs the intestinal health status in several carnivorous fish species, accompanied with poor nutrient digestibility [17–26]. Our previous work also revealed that dietary soybean meal induced intestinal enteritis and reduced the digestive and absorptive functions in turbot [27,28]. Nevertheless, it is still unclear whether the low digestibility of CGM in turbot is caused by the influence of CGM on the intestinal health status, and few reports are available on this subject.

The present work was conducted to elucidate the causes of the poor utilization of CGM by turbot from the view of intestinal health. For this purpose, we evaluated the effects of CGM on the growth performance, nutrient digestibility, and intestinal health status, determined though the assessment of indicators, including intestinal morphology, inflammatory responses, immunity, and redox homeostasis.

## Materials and methods

### Ethic statement

The usage of fish was in strict accordance with the recommendations of the Guidelines for the Use of Experimental Animals of Shandong University. The protocol for animal care and handling used in this study was approved by the Animal Experimental Ethics Inspection of Shandong University (Permit Number: 20170313). Before sacrificing and handling, experimental fish were anesthetized with 100 ng/ml ethyl 3-aminobenzoate methanesulfonic acid (MS222, Sigma, USA) and all Efforts were made to minimize uneasiness of animals during all processes.

### Feed ingredients and diet formulation

Based on previous work [27], a fish meal based diet (FM diet) was formulated to contain 48% crude protein and 12% crude lipid with fish meal as the primary protein source, fish oil and soybean oil as lipid sources, and wheat flour as the carbohydrate source (Table 1). This diet was utilized as the control diet. Based on the FM diet, another three isonitrogenous and isolipidic diets were formulated to contain 212 g kg^−1^, 318 g kg^−1^, and 426 g kg^−1^ CGM as replacement of 33%, 50%, and 67% fish meal protein in the basal diet, which were named CGM20, CGM30, and CGM40, respectively (Table 1). The inclusion level of CGM was set according to the recommendations of Regost *et al* [13]. Crystalline amino acids (lysine, arginine, and tryptophan) were supplemented to meet the essential amino acid requirements of turbot [29,30] or maintain the tryptophan level in the diets (Table 1 and Table 2). The diet preparation and storage were performed as described in our previous work [31]. Standard methods were used to analyze the feed ingredients and experimental diets [32]. The dry matter and ash contents were determined gravimetrically to constant weight in an oven at 105°C and 550°C, respectively. Crude lipid content was determined gravimetrically after extraction with ethyl ether (Buchi extraction system B-811, Buchi Labortechnik AG, Switzerland). Crude protein was determined by the Kjeldahl method with a FOSS Kjeltec System (Kjeltec 2300 Distillation Unit, Foss Tecator, Höganäs, Sweden) using boric acid to trap the released ammonia. Gross energy was determined by adiabatic bomb calorimeter (PARR1281, Parr Instrument Company, Moline, Illinois, USA). For determination of the essential amino acids (except for methionine), the samples from the feed ingredients and the prepared diets were freeze-dried and hydrolyzed with 6 N HCl at 110°C for 24 h, followed by analysis with a Biochrom 30 amino acid analyzer (Biochrom Ltd, Cambridge, Science Park, England); Methionine was determined using reverse-phase high-performance liquid chromatography (HP1100, Agilent Technologies, Wilmington, DE, USA) [10]. The starch level in the diets was determined by the amylase/amyloglucosidase method using a Starch Assay Kit from Sigma (St. Louis, MO, USA; Product Code STA20). Yttrium oxide (Y_2_O_3_) was utilized as an inert tracer in each diet for determining ADCs, and its content was established by an inductively coupled plasma-atomic emission spectrophotometer (ICP–AES, Vista-MPX, Varian Instruments, Walnut Creek CA, USA) after perchloric acid digestion.

**Table 1:** Ingredients and compositions of experimental diets (dry-matter basis).

	Experimental diet^1^			
	FM	CGM20	CGM30	CGM40
*Ingredients (g kg*^*-1*^*)*				
Fish meal ^a^	620	405	300	182
Corn gluten meal ^b^	0	212	318	426
Wheat meal	245	245	245	245
Fish oil	17	38	49	60
Soybean oil	17	13	11	9
Soybean lecithin	20	20	20	20
Vitamin and mineral premix ^c^	25	25	25	25
Monocalcium phosphate	0	10	10	10
Choline chloride	5	5	5	5
Calcium propionic acid	1	1	1	1
Yttrium oxide	1	1	1	1
Ethoxyquin	1	1	1	1
Arginine	0	1	2	4
Lysine	0	3	6	9
Tryptophan	0	1	2	2
Cellulose	48	19	4	0
*Proximate composition (%)*				
Dry matter	95.12	94.98	95.13	94.88
Crude protein	48.13	48.06	48.26	48.04
Crude lipid	12.31	12.22	12.21	12.11
Starch	20.09	24.11	26.13	28.18
Ash	12.88	9.95	8.78	6.30
Gross energy (KJ/g)	20.42	21.17	21.58	21.89

^1^ FM: a basal diet; CGM20, about 20% of the corn gluten meal inclusion level to replace 33% fish meal protein in basal diet; CGM30, about 30% of the corn gluten meal inclusion level to replace 50% fish meal protein in basal diet; CGM40, about 40% of the corn gluten meal inclusion level to replace 67% fish meal protein in basal diet.

^a^ Fish meal: steam dried fish meal (COPENCA Group, Lima, Peru)

^b^ Corn gluten meal, purchased from Qingdao Greatseven Co. Ltd

^c^ Vitamin premix supplied the diet with (mg kg^−1^ diet) the following compounds: retinyl acetate, 32; vitamin D_3_, 5; DL-α-tocopherol acetate, 240; vitamin K_3_, 10; thiamin, 25; riboflavin (80%), 45; pyridoxine hydrochloride, 20; vitaminB_12_ (1%), 10; Lascorbyl-2-monophosphate-Na (35%), 2000; calcium pantothenate, 60; nicotinic acid, 200; inositol, 800; biotin (2%), 60; folic acid, 20; cellulose, 11473. Mineral premix consisted of (mg kg^−1^ diet) the following ingredients: FeSO_4_·H_2_O, 80; ZnSO_4_·H_2_O, 50; CuSO_4_·5H_2_O, 10; MnSO_4_·H_2_O, 45; KI, 60; CaCl_2_·6H_2_O (1%), 50; Na_2_SeO_3_ (1%), 20; MgSO_4_·7H_2_O, 1200; zoelite, 8485.

**Table 2:** Essential amino-acid profile of the diets and requirements of turbot (g 16 g^-1^ N).

	Experiment diet				EAA requirements	
Amino acid	FM	CGM20	CGM30	CGM40	1	2
Threonine	3.04	3.11	3.11	3.11	2.9	2.37
Phenylalanine	3.14	3.93	4.27	4.64	5.3*	2.54
Lysine	5.70	5.15	5.12	5.06	5.0	5.00
Valine	4.26	4.26	4.21	3.77	2.9	2.74
Leucine	6.21	9.36	10.81	12.35	4.6	4.47
Isoleucine	3.34	3.40	3.39	3.38	2.6	2.59
Methionine	2.95	2.94	2.90	2.85	2.7^#^	1.68
Arginine	4.69	4.27	4.26	4.24	4.8	4.22
Histidine	1.61	2.32	2.64	2.98	1.5	1.28
tryptophan	1.06	1.08	1.07	1.06	Not determined	

^1^ From Kaushik [29].

^2^ From Peres & Oliva-Teles [30].

* Phenylalanine + tryptophan

^#^ Methionine + cysteine

### Experimental procedures

Apparent disease-free juvenile turbot were obtained from a commercial farm in Haiyang, China, and transferred to an indoor flow-through water system in the Haiyang Yellow Sea Aquatic Product Co., Ltd (Yantai, China). The fish were acclimated to the system and fed the FM diet for two weeks. Next, turbot individuals with an initial body weight of approximately 11.4 g were randomly distributed and transferred into 12 tanks, 30 fish per tank. Each tank was filled with 300 L of seawater. The seawater was pumped from the adjacent coastal water, filtered through a sand filter, and distributed to each tank at a rate of approximately 2.0 L/min. Each diet was fed to fish in three tanks. Fish were fed the experimental diets twice daily (at 07:00 and 18:00) to apparent satiation, and the feed consumption was recorded. During the 8-week feeding trial, the water temperature was 12°C–16°C, pH was 7.8–8.2, and the salinity was 28–30 ‰.

### Sampling

After eight weeks of feeding, the feces were collected following methods commonly applied for turbot digestibility determination [33–35]. The feces were siphoned with an automatic feces collector after 2–4 h feeding. The pooled feces samples within each replicate were dried for 12 h at 50°C and stored at -20°C. The dry matter, crude protein, and Y_2_O_3_ contents of the feces samples were determined as described before.

After a sufficient number feces samples (more than 5 g, dry matter) were collected, all experimental fish were anesthetized with eugenol (1: 10,000, Shanghai Reagent Co., Shanghai, China) and their body weights were recorded before sampling. Then, eight fish from each tank were randomly selected, and blood samples were collected from the caudal vein using heparinized syringes. Plasma samples were obtained by centrifugation (4,000 × g for 10 min) at 4°C and immediately stored at -80°C until analysis. Subsequently, the fish were killed with a blow to the head, and their body lengths were determined. The intestine was removed, cleared from any mesenteric, adipose tissue, and rinsed with ice-cold phosphate buffer saline to remove the eventual remaining gut contents. Only fish with food in the process of digestion in the intestinal tract were sampled to ensure recent intestinal exposure to the diets. Four of the eight sampled fish were randomly selected, and their distal intestines (DI) were removed individually and divided into three parts for histological and gene expression examination. A part of the DI of each fish was placed in 4% phosphate-buffered formaldehyde solution for 24 h and subsequently stored in 70% ethanol until further processing for light microscopy analysis. The second part was fixed in 2.5% glutaraldehyde in 0.1 M phosphate buffer (pH 7.0) for transmission electron microscopy (TEM) analysis. The fixed samples were washed in buffer, dehydrated with graded ethanol, and embedded in Spurr’s resin for ultrastructure observation. The third part for gene expression analysis was placed in RNAlater (Ambion, Austin, TX, USA), stored at 4°C for 24 h, then temporarily at -20°C, and finally at -80°C. In these three analyses, the DI from one fish was treated and stored individually and assigned a specific code. For enzyme activity assessment, the intestinal tissues sampled from the mid-intestine (MI) to DI from the other four fish per tank were frozen in liquid N_2_ and stored at -80°C.

### Histology

Fixed DI tissue samples were processed according our previous work and stained with hematoxylin and eosin (H&E)[27]. Examination was conducted blindly with a light microscope using a continuous scoring scale from 0 to 10 as described by Penn *et al*. [36]. The following histological properties were evaluated: length and fusion of mucosal folds, cellular infiltration and width of the lamina propria, and submucosa and enterocyte vacuolization.

The DI samples from FM and CGM40 groups for TEM analysis were processed as follows: one ultrathin section for each intestinal sample (a total number of 12 intestine samples per dietary treatment) was cut and stained with 2% uranyl acetate, post-stained with 0.2% lead citrate, and examined in a JEOL-JEM 1200 TEM (JEOL, Tokyo, Japan) at 80 kV. All digital images were captured with Olymbus SIS software and analyzed using Image J version 1.36.

### Quantitative real-time PCR (qPCR)

Total RNA was extracted and purified from DI tissue samples (approximately 50 mg) using the RNeasy Protect Mini Kit RNeasy Protect Mini Kit (Qiagen, GmbH, Hilden, Germany; Product Code 74126) according to the manufacturer's instructions. Next, RNA was quantified using a NanoDrop ND-1000 spectrophotometer (Nano-Drop Technologies, Wilmington, DE, USA), and its quality was checked by Agilent Bio-Analyzer (Agilent Technologies, Santa Clara, CA, USA). The cDNA synthesis was performed with the QuantiTect Reverse Transcription Kit (Qiagen, GmbH, Hilden, Germany; Product Code 205311) using 1.0 μg of RNA, following the manufacturer indications.

The expression profiles of the genes of interleukin-1 beta (*Il-1β*), interleukin 8 (*Il-8*), tumor necrosis factor α (*Tnf-α*), and the transforming growth factor β (*Tgf-β*) were determined using real-time quantitative PCR (RT-qPCR) with ribosomal protein S4 (RPS4) utilized as the house-keeping gene for sample normalization. The experiment was conducted as described in our previous work [37].

### Serum diamine oxidase activity and D-lactate level assay

As detailed in our previous study [38], serum diamine oxidase activity and D-lactate level were measured using a Diamine Oxidase (DAO) Assay Kit (Nanjing Jiancheng Bioengineering Institute, Nanjing, Jiangsu, China; Product Code A088-1) and D-Lactic Acid ELISA kit (Nanjing Jiancheng Bioengineering Institute, Nanjing, Jiangsu, China; Product Code H263), respectively.

### Intestinal biochemical analysis

Intestinal samples (MI to DI) were homogenized in 10 volumes (w/v) of ice-cold physiological saline and centrifuged at 5,000 g for 20 min at 4°C. Then, the supernatants were collected, equally divided into 20 pieces, and stored at -80°C until use.

The activities of three brush-border enzymes, including maltase (MAL), leucine aminopeptidase (LAP), and alkaline phosphatase (AKP), four antioxidant enzyme, including superoxide dismutase (SOD), catalase (CAT), glutathione peroxidase (GPX), and glutathione reductase (GR), and lysozyme (LZM), as well as the concentrations of complement 3 (C3), complement 4 (C4), immunoglobulin M (IgM), and the intestinal reduced glutathione (GSH), were assayed according to the methods and procedures described in our previous publications [27,37,38]. The intestinal malondialdehyde (MDA) level as lipid peroxidation indication was determined by a Malondialdehyde (MDA) Assay Kit (Nanjing Jiancheng Bioengineering Institute, Nanjing, Jiangsu, China; Product Code A003-1) [39]. The intestinal acid phosphatase (ACP) activity was determined using an Acid Phosphatase Assay Kit (Nanjing Jiancheng Bioengineering Institute, Nanjing, Jiangsu, China; Product Code A060-1) [40–42].

### Calculations and statistical analysis

Growth performance and feed utilization were assessed based on the specific growth rate and feed efficiency ratio. The specific growth rate (SGR) was calculated using the tank means for initial body weight (IBW) and final body weight (FBW), and calculated as follows:

SGR = [(ln FBW − ln IBW)/number of days] × 100 and calculated as follows:

Feed efficiency ratio was calculated as:

Feed efficiency ratio (FER) = (FBW − IBW) / total amount of the feed consumed.

The apparent digestibility coefficients for dry matters (ADCd) were calculated as follows:

ADCd (100%) = (1- (Y_2_O_3_% in diet)/ (Y_2_O_3_% in feces)) ×100%.

The apparent digestibility coefficients for protein (ADCp) were calculated as follows:

ADCp (100%) = (1-(Y_2_O_3_% in diet)/ (Y_2_O_3_% in feces)) ×(%protein in feces /%protein in diet) ×100%

The effects of the inclusion levels of CGM were evaluated using regression analysis. The results were fit to polynomial models of the first and second degrees. The model was considered to best fit the results on the basis of visual examination and the observed R^2^ is reported. One way ANOVA (analysis of variance) was applied to analysis data other than light microscopy and Duncan's multiple range tests was applied to test differences between the means. The level of significance was set as *P* < 0.05. The light microscopy analysis was performed using the Wilcoxon/Kruskal-Wallis test followed by the posthoc Wilcoxon method to compare the means. To evaluate the TEM data, the differences among the means were analyzed by t-test. The level of significance was set at *P* < 0.05.

## Results

### Effect of corn gluten meal on growth performance, digestibility, and nutrient utilization in turbot

**Fig 1:**
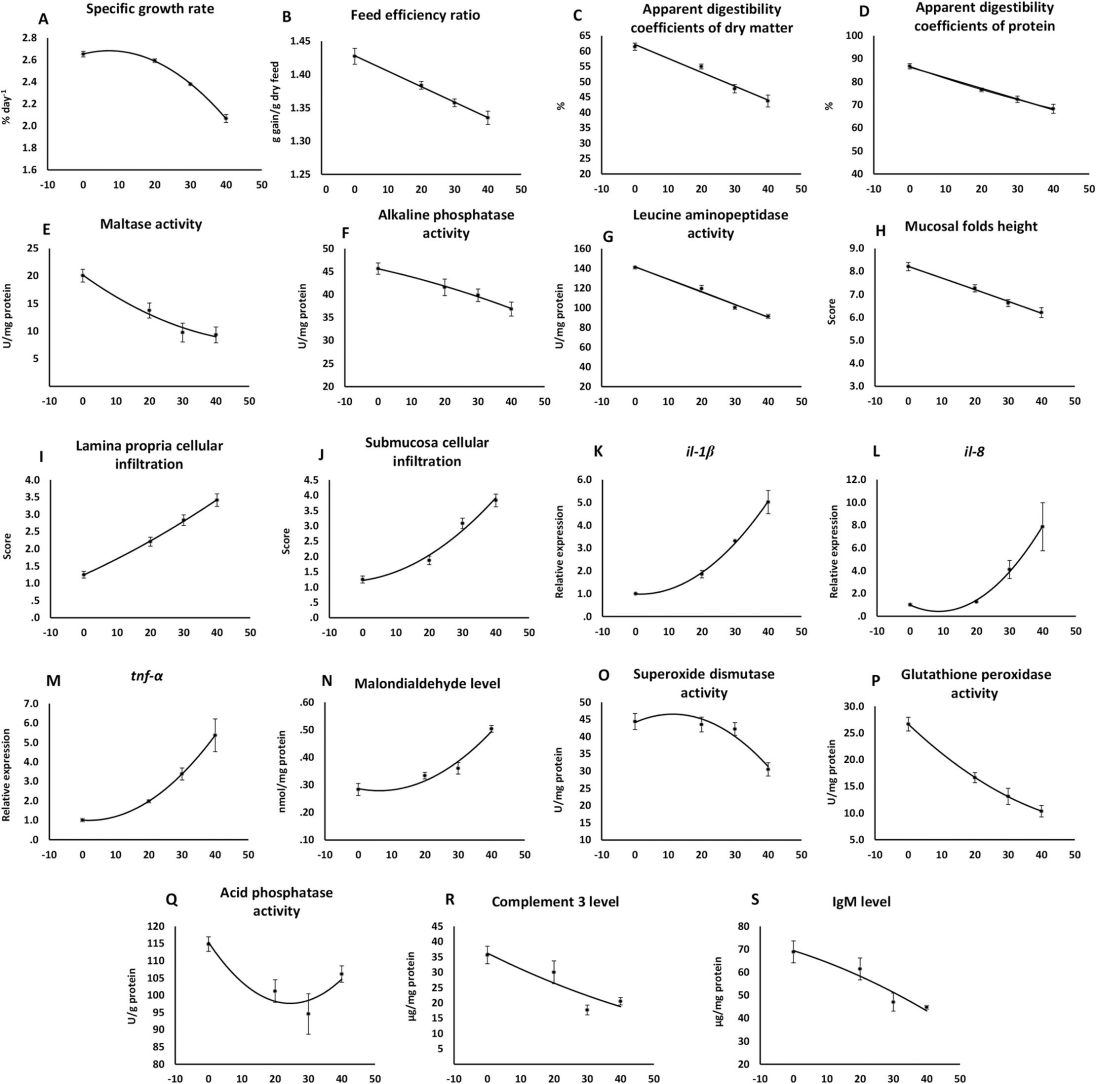
**Illustration of the relationships between the level of dietary corn gluten meal supplementation (%) and (A) the specific growth rate (SGR; % per day), (B) feed efficiency ratio, (C) apparent digestibility coefficients of dry matter (%), (D) apparent digestibility coefficients of protein (%), (E) maltase activity (U/mg protein), (F) alkaline phosphatase activity (U/mg protein), (G) leucine aminopeptidase (U/mg protein), (H) mucosal folds height in DI (score), (I) lamina propria cellular infiltration in DI (score), (J) submucosa cellular infiltration in DI (score), (K) mean normalized expression of *Il-1β* in DI tissue, (L) mean normalized expression of *Il-8* in DI tissue, (M) mean normalized expression of *Tnf-α* in DI tissue, (N) intestinal malondialdehyde level (nmol/mg protein), (O) intestinal superoxide dismutase activity (U/mg protein), (P) intestinal glutathione peroxidase activity (U/mg protein), (Q) intestinal acid phosphatase activity (U/g protein), (R) intestinal complement 3 level (μg/mg protein) and (S) intestinal IgM level (μg/mg protein).** The curves illustrate the regression that fits the results best according to the regressions shown in Table 3. DI: distal intestine.

During the feeding trial no mortality was recorded. The effects of the experimental diets on turbot growth performance, feed utilization, and digestibility of dry matter and protein are presented in Table 3, S1 Table and Fig 1A–1D. The regression analysis results showed a significant inverse relationship between growth and CGM level following a second-degree relationship. The increased level of CGM reduced those of FER, ADCd, and ADCp following a first-degree relationship.

**Table 3:** Results of regression analysis of effects of increasing doses of corn gluten meal on growth performance, feed utilization, histology, cytokines gene expression, intestinal permeability, oxidant and antioxidant indices, and immune parameters data of turbot.

	P (model)	R^2^	intercept	X	X^2^
Specific growth rate	<0.001	0.97	2.6547	0.0083	-0.0006
Feed efficiency ratio	<0.001	0.87	1.4283	-0.0023	
Apparent digestibility coefficients of dry matter (%)	<0.001	0.91	62.127	-0.4519	
Apparent digestibility coefficients of protein (%)	<0.001	0.93	86.384	-0.4627	
Maltase activity (U/mg protein)	<0.001	0.8	20.176	-0.4318	0.0038
Alkaline phosphatase activity (U/mg protein)	0.005	0.69	45.596	-0.1618	-0.0013
Leucine aminopeptidase (U/mg protein)	<0.001	0.94	141.58	-1.276	
Mucosal folds height	<0.001	0.62	8.2219	-0.0509	
Mucosal folds fusion	<0.001	0.64	1.9261	0.0162	0.0007
Lamina propria width	<0.001	0.58	1.5788	0.0298	0.0004
Lamina propria cellular infiltration	<0.001	0.74	1.2462	0.0439	0.0003
Submucosa width	<0.001	0.66	1.4636	-0.0261	0.0018
Submucosa cellular infiltration	<0.001	0.76	1.2212	0.0152	0.0013
Enterocyte vaculization	<0.001	0.5	8.0568	-0.0258	-0.0002
Enterocyte nucleus position	<0.001	0.59	1.8803	-0.0011	0.0001
*Il-1β* expression	<0.001	0.94	0.9887	-0.0075	0.0027
*Il-8* expression	0.002	0.75	0.9881	-0.1307	0.0076
*Tnf-α* expression	<0.001	0.87	0.003	-0.0118	0.003
*Tgf-β* expression	0.002	0.76	1.0901	0.0306	0.0011
Serum diamine oxidase activity	0.529	0.13	10.437	-0.0339	
Serum D-lactate level	0.305	0.23	11.353	-0.0553	
Malondialdehyde level (nmol/mg protein)	<0.001	0.88	0.2865	-0.0024	0.0002
Superoxide dismutase activity (U/mg protein)	0.002	0.76	44.428	0.4218	-0.0186
Catalase activity (U/mg protein)	<0.001	0.82	4.061	-0.0417	0.0002
Reduced glutathione level (mg/g protein)	0.002	0.73	15.29	-0.231	0.0021
Glutathione peroxidase activity (U/mg protein)	<0.001	0.92	26.662	-0.5906	0.0046
Glutathione reductase activity (U/g protein)	0.004	0.72	4.6113	-0.0425	
Lysozyme (**μ**g/mg protein)	0.474	0.15	0.0467	0.0002	
Acid phosphatase activity (U/g protein)	0.02	0.58	115.34	-1.4437	0.0294
Complement 3 level (**μ**g/mg protein)	0.008	0.65	36.236	-0.5386	0.0026
Complement 4 level (**μ**g/mg protein)	0.028	0.59	19.404	-0.2186	0.0006
IgM level (**μ**g/mg protein)	0.004	0.71	69.408	-0.4562	-0.005

Abbreviations: *Il-1β*: interleukin-1 beta; *Il-8*: interleukin 8; *Tnf-α*: tumor necrosis factor α; *Tgf-β*: transforming growth factor β.

### Effect of corn gluten meal on the brush-border membrane enzyme activities in turbot

The data of the enzymatic activities of MAL, AKP, and LAP presented as specific activities are shown in Table 3, S1 Table and Fig 1E–1G. The increase in the dietary CGM levels significantly decreased the specific activities of all the three tested brush-border membrane enzymes. The relationship followed a second-degree function in MAL and AKP activities and a first-degree function in LAP activity.

### Effect of corn gluten meal on the light microscopic structure of the distal intestine in turbot

**Fig 2:**
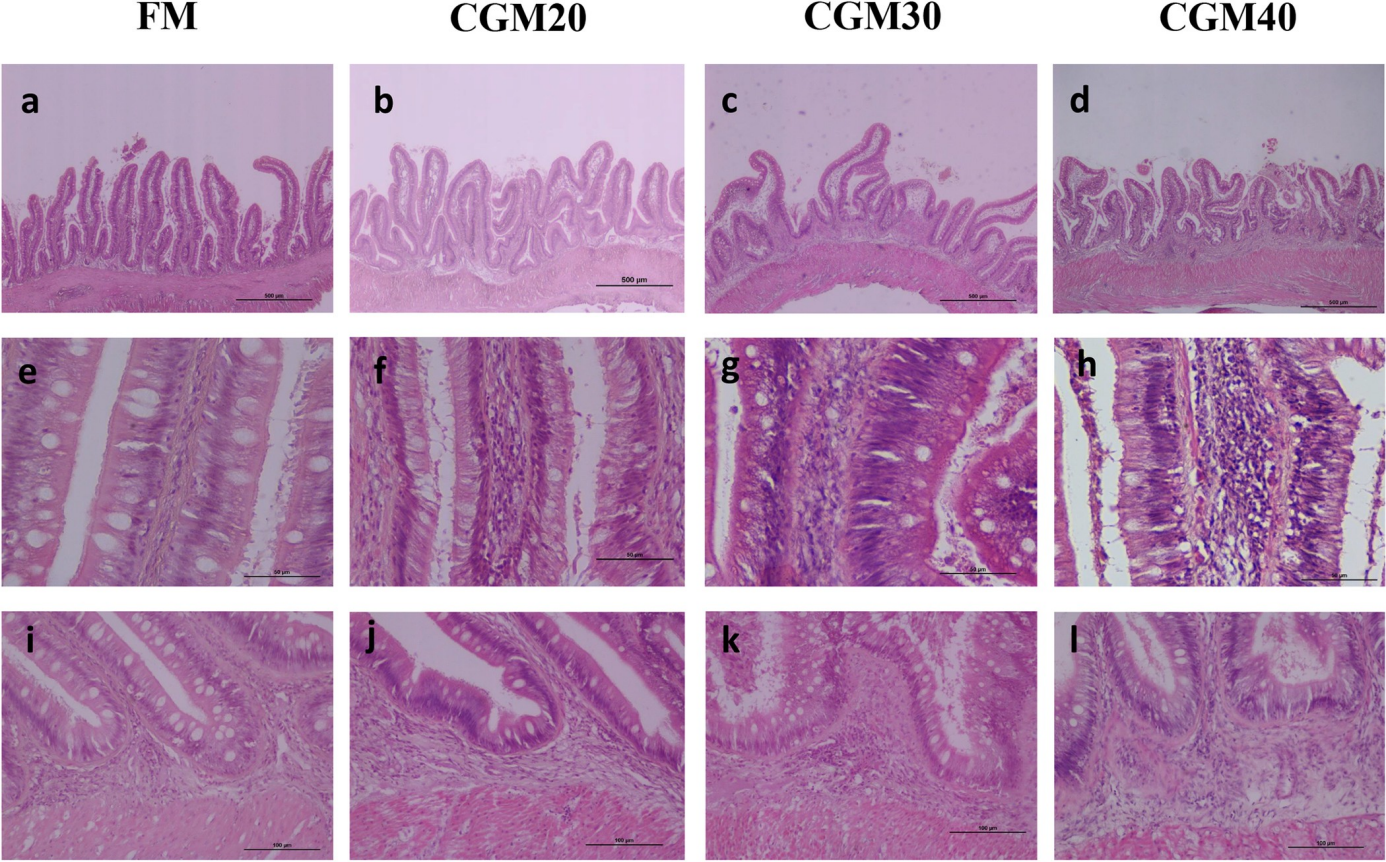
**Representative histomorphological images of hematoxylin and eosin-stained sections of the distal intestine of turbot depicting the gradual increase in the severity of the inflammatory changes with increasing corn gluten meal supplementation in turbot fed the FM (a, e, and i), CGM20 (b, f, and g), CGM30 (c, g, and k) and CGM40 (d, h, and l).** (a–d) Representative images of decreased height and increased fusion of the mucosal folds with increasing corn gluten meal level (bar = 500 **μ**m). (e–h) Representative images of the increased width and cellular (leucocyte) infiltration of the lamina propria with the increase in the corn gluten meal level and images of reduced numbers of supranuclear absorptive vacuoles in enterocytes (bar = 50 **μ**m). (i–l) Representative images of increased width and cellular (leucocyte) infiltration of the submucosa with increasing corn gluten meal level (bar = 100 **μ**m). FM: a basal diet; CGM20, about 20% of the corn gluten meal inclusion level to replace 33% fish meal protein in basal diet; CGM30, about 30% of the corn gluten meal inclusion level to replace 50% fish meal protein in basal diet; CGM40, about 40% of the corn gluten meal inclusion level to replace 67% fish meal protein in basal diet.

The inclusion level of corn gluten meal affected all characteristics assessed and induced alterations typical for mucosal inflammation (Fig 2). The severity increased with rise in the level of CGM inclusion. The inclusion of CGM decreased the height and elevated the fusion of the mucosal folds. It also increased the width and cellular (leucocyte) infiltration of the lamina propria and submucosa and reduced the numbers of supra-nuclear absorptive vacuoles in enterocytes (S1 Table). With the increase in the CGM level, the mucosal folds height followed a first-degree relationship, whereas the other characteristics followed a second-degree relationship (Table 3 and Fig 1H–1J).

### Effect of corn gluten meal on the gene expression of the distal intestinal cytokines in turbot

As can be seen from the Table 3, S1 Table and Fig 1K–1M, the expression levels of *Il-1β*, *Il-8*, *Tnf-α*, and *Tgf-β* showed gradual and accelerating increases with the rise in the CGM level in the diet, fitting a second-degree relationship.

### Effect of corn gluten meal on the electron microscopic structure of the distal intestine in turbot

**Fig 3:**
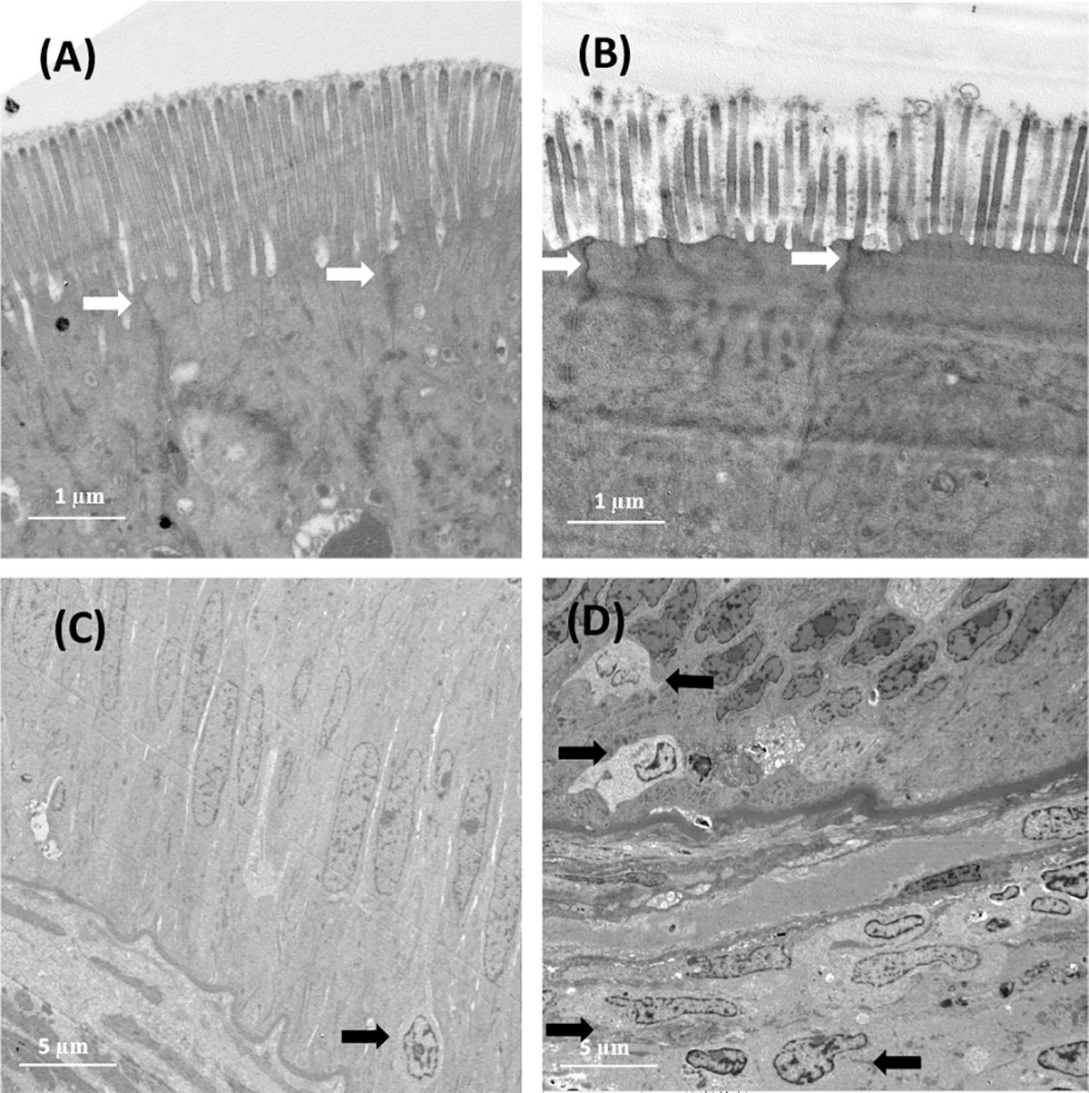
Electron microscopic structure of the distal intestine epithelium of turbot fed experimental diets. (A) and (C), FM diet, a basal diet; (B) and (D), CGM40 diet, approximately 40% of the corn gluten meal inclusion level to replace 67% of the fish meal in the FM diet. In comparison to ultrastructure of figure (A), that of the samples in figure (B) had shorter and less dense microvilli. The white arrow in (A) and (B) represents apical tight junction (TJ). No significant difference was observed in TJ structure between figure (A) and figure (B). The black arrow in (C) and (D) represents the infiltrated leucocytes from the submucosa into the epithelium layer.

The TEM data revealed that dietary CGM considerably changed the structure of the distal intestinal microvilli (Fig 3A and 3B). The fish fed the CGM40 diet showed significantly shorter and less dense microvilli than those fed the FM diet (Table 4). No significant difference was observed in the tight junction structure between the fish fed the FM diet and that fed the CGM40 diet (Fig 3A and 3B). The TEM analysis results showed that all 12 fish individuals fed the CGM40 diet exhibited higher infiltration of leucocytes from the submucosa to the epithelium layer than that in the fish fed the control diet (Fig 3C and 3D).

**Table 4:** Microvillar morphology of turbot fed with different experiment diets (n = 12).

	FM	CGM40	Pooled S.E.	*P* value
Microvilli length (**μ**m)	2.41^a^	1.81^b^	0.05	<0.001
Microvilli density^1^	16.6^a^	11.5^b^	0.52	<0.001

Different letters in the same row mean significant differences (*p*<0.05). FM, the control diet; CGM40, about 40% of the corn gluten meal inclusion level to replace 67% fish meal in basal diet.

^1^ Microvilli density was expressed as the number of microvilli in a 2-**μ**m standardized region on the surface of enterocyte.

### Effect of corn gluten meal on the intestinal epithelial permeability in turbot

As can be observed in Table 3 and S1 Table, the increasing level of CGM caused no significant difference in the serum DAO activity or D-lactate level.

### Effect of corn gluten meal on the intestinal oxidant and antioxidant indices in turbot

The data of intestinal oxidant and antioxidant indices are shown in Table 3, S1 Table and Fig 1N–1P. The regression analysis showed that the oxidant indices of MDA significantly increased with the rise in the level of CGM, and the antioxidant indices of SOD, CAT, GPX, and GR activities and the GSH level showed the opposite changes after the CGM inclusion. All these indices fit a second-degree relationship best.

### Effect of corn gluten meal on the intestinal immune parameters in turbot

The data of intestinal immune parameters are shown in Table 3, S1 Table and Fig 1Q–1S. The intestinal ACP activity, C3 and C4 levels, and IgM level significantly decreased with the increasing level of CGM, fitting a second-degree relationship. No significant difference in LZM activity with the rise in the level of CGM.

## Discussion

The findings of the present work showed that the replacement of fish meal with CGM negatively influenced the growth performance, nutrient digestibility, and feed utilization in turbot. Our results are consistent with those obtained by Regost *et al*. [13]. In the present study, CGM supplementation negatively influenced the activities of brush-border membrane enzymes, which are responsible for the final stage of luminal digestion prior to absorption [43]. Generally, the activities of brush-border membrane enzymes can reflect the digestive capacity. Previous examinations found that the supplementation of plant protein sources in carnivorous fish significantly decreased these activities [27,36,44–50]. Thus, the decreased nutrient digestibility and final body weight in CGM-fed fish could be partly attributed to the negative effects of CGM on brush-border membrane enzymes.

The present study results clearly demonstrated that dietary CGM caused a dose-dependent increase in the severity of inflammatory changes in DI tissue in turbot. Dietary CGM increased the width and cellular infiltration in lamina propria and submucosa, and induced higher expression of pro-inflammatory cytokine genes, including *Il-1β*, *Il-8*, and *Tnf-α*. This findings is similar to our previous results concerning soybean meal-induced enteritis (SBMIE) in turbot [27]. The inflammatory responses in CGM40 fed fish were the strongest as shown by the highest degree of cellular infiltration in both lamina propria and submucosa, and the expression of pro-inflammatory cytokines, which was in agreement with the TEM data showing obvious infiltration of leukocytes in the epithelial monolayer. Moreover, the CGM40 diet changed the microvilli structures. A similar phenomenon was also observed in turbot fed a high dietary level of SBM [28]. However, differently from previously obtained data for SBMIE in turbot [28,38], the CGM changed neither the serum DAO activity or D-lactate level nor the intercellular junction of turbot as established by TEM data. DAO is the an intracellular enzyme mainly exists in the intestinal mucosa [51] and D-lactate is a metabolic product of intestinal bacteria [52]. Serum DAO activity or D-lactate level are indices of intestinal wall permeability because intact intestinal mucosa prevents DAO and D-lactate infiltrating into the portal blood [53]. The studies conducted by our team on turbot [38] as well as those performed by other researchers on Atlantic salmon [54,55] revealed that soya-saponins are the key factors that induce SBMIE probably by destructing the cellular junction structure and increasing the intestinal permeability. All these results indicate that CGM may have a different way, at least not by changing cellular junction structure and intestinal permeability, to induce inflammatory changes from that of the soybean meal.

The intestinal immunity and redox homeostasis play pivotal roles in maintaining intestinal health in fish [56]. The intestine of teleost has various important defense molecules, such as lysozymes, complement systems, phosphatases, and immunoglobulins, which participate in its protection against microbial pathogens [57]. In the present study, we observed negative effects of CGM on the intestinal immunity of CGM-fed fish revealed by the decreased activities of ACP and the C3, C4, and IgM levels in a dose-dependent manner, which is similar to the impairment caused by a soybean meal on the immune system in several finfish species [58]. Redox homeostasis is the balance between reactive oxygen species production and elimination [59].The fish intestine has high content of polyunsaturated fatty acids (up to 24.9% of the total fatty acid composition), which increases its susceptibility to the negative effects of reactive oxygen species [60]. The imbalance in the redox homeostasis in the fish intestine results in considerable lipid peroxidation, expressed by the increased MDA content [61]. In the present work, the intestinal level of MDA increased with the rise in the CGM level, suggesting that CGM supplementation causes redox homeostasis imbalance and induces intestinal oxidative injury in turbot. Antioxidant defense mechanisms involve enzymes and non-enzymes that maintain the redox homeostasis. The superoxide radical anion undergoes dismutation by SOD and generates hydrogen peroxide, which can be transformed into water by CAT and GPx converting GSH to its oxidized form. The presence of GR is responsible for the regeneration of GSH [62]. In the present work, all these antioxidant parameters decreased with the increase in the CGM inclusion, which indicates that CGM impairs the antioxidant system activities of the turbot intestine. Similar results were obtained in yellow catfish fed high inclusion of soybean meal [63]

In CGM, the non-protein material, mainly carbohydrates, accounts for more than 30% [3]. The starch level in CGM ranges from 13% to 20.7% [64,65]. A substitution fish meal with CGM can increase the digestible carbohydrate level in the diet. In the present work, the starch level increased with rise in the CGM level in the diet. More specifically, an elevation from 20.02% in the control diet to 28.18% in the CGM40 diet was observed. The optimal dietary digestible carbohydrate level is less than 20% for carnivorous fish, such as gilthead sea bream [66], golden pompano[67], giant croaker [68], and Chinese long snout catfish [69]. Furthermore, the maximum recommended levels of dietary carbohydrate inclusion fall within 15%–25% for marine fish [70]. These data indicate that the substitution of a fish meal with CGM in the present work increased dietary carbohydrate levels to high extremes. Carnivorous fish have been shown to have a low ability to utilize carbohydrates [71]. Furthermore, previous results showed that the excessive dietary carbohydrate impaired the immunity and redox homeostasis of carnivorous fish, such as golden pompano [72], largemouth bass [73], yellow catfish [74], and black carp [75], and caused inflammation in the liver of Japanese flounder [76]. However, insufficient research has been conducted on the dietary carbohydrates in the fish intestine. Castro *et al*. discovered that a carbohydrate-rich diet negatively affected the GSH redox status in the intestine of seabass [77]. All these results suggest that excessive carbohydrates from CGM is at least one of the major factors causing the damage in the immunity and redox homeostasis, and even inducing inflammatory changes in turbot fed CGM, although this notion requires validation in future studies.

Except the excessive carbohydrate level, imbalanced amino-acid content is another drawback of CGM as a protein source compared with fish meal. CGM is deficient in lysine and arginine but high in leucine [3]. In the present work, crystal lysine and arginine were supplemented to satisfy the nutrient requirements of the fish. However, the leucine level increased dramatically with the rise in the CGM inclusion and was much higher than the turbot requirement. Leucine is an independent amino acid in fish, but the high level of dietary leucine was found to be harmful to fish growth performance [78,79]. The effects of excessive leucine on the intestinal health have been deeply investigated in grass carp. Specifically, the high levels of dietary leucine caused decreases in LZM and ACP activities and the C3 content, up regulated the pro-inflammation cytokine IL-8 mRNA level, and decreased the GSH content and the activities of CuZnSOD and GPx in the intestine of grass carp [78,80]. Thus, the high leucine content of CGM might be another factor that affects the intestinal health in turbot that could not be ignored.

In conclusion, CGM exerted negative effects on the intestinal health in turbot, including the induction of enteritis in the DI tissue and impaired intestinal immune and antioxidative systems. CGM may induce enteritis in a way that is different from that of soybean meal since the cellular tight junction structure and the intestinal permeability were not affected by its inclusion. The present work directly suggests that strategies to maintain intestinal health should be developed and undertaken when formulating turbot diets containing CGM.

## Supporting information

S1 Table Results of one-way ANOVA analysis of effects of corn gluten meal on growth performance, feed utilization, histology, cytokines gene expression, intestinal permeability, oxidant and antioxidant indices, and immune parameters data of turbot. Data were expressed as mean values with the standard errors. Data in the same row with different superscript letters are significantly different (*P*< 0.05). Abbreviations: FM, a basal diet; CGM20, about 20% of the corn gluten meal inclusion level to replace 33% fish meal protein in basal diet; CGM30, about 30% of the corn gluten meal inclusion level to replace 50% fish meal protein in basal diet; CGM40, about 40% of the corn gluten meal inclusion level to replace 67% fish meal protein in basal diet. *Il-1β*, interleukin-1 beta; *Il-8*, interleukin 8; *Tnf-α*, tumor necrosis factor α; *Tgf-β*, transforming growth factor β.
(DOCX)
