# Supplementary material for: Corn gluten meal induces enteritis and decreases intestinal immunity and antioxidant capacity in turbot (Scophthalmus maximus) at high supplementation levels
Source: PLoS One. 2019 Mar 13;14(3):e0213867. doi: 10.1371/journal.pone.0213867 (PMC6415862; doi:10.1371/journal.pone.0213867)
Supplement: S1 Table — Data were expressed as mean values with the standard errors. Data in the same row with different superscript letters are significantly different (P< 0.05). Abbreviations: FM, a basal diet; CGM20, about 20% of the corn gluten meal inclusion level to replace 33% fish meal protein in basal diet; CGM30, about 30% of the corn gluten meal inclusion level to replace 50% fish meal protein in basal diet; CGM40, about 40% of the corn gluten meal inclusion level to replace 67% fish meal protein in basal diet. Il-1β, interleukin-1 beta; Il-8, interleukin 8; Tnf-α, tumor necrosis factor α; Tgf-β, transforming growth factor β. (DOCX) [file pone.0213867.s001.docx]

**S1 Table.** **Results of one-way ANOVA analysis of effects of corn gluten meal on growth performance, feed utilization, histology, cytokines gene expression, intestinal permeability, oxidant and antioxidant indices, and immune parameters data of turbot**.

|  | FM | CGM20 | CGM30 | CGM40 | *P-*value |
| --- | --- | --- | --- | --- | --- |
| Specific growth rate | 2.65±0.03^c^ | 2.60±0.02^c^ | 2.38±0.01^b^ | 2.07±0.04^a^ | <0.001 |
| Feed efficiency ratio | 1.43±0.01^c^ | 1.38±0.01^b^ | 1.36±0.01^bc^ | 1.34±0.01^a^ | <0.001 |
| Apparent digestibility coefficients of dry matter (%) | 61±1.2^c^ | 55±0.8^b^ | 48±1.4^a^ | 44±1.9^a^ | <0.001 |
| Apparent digestibility coefficients of protein (%) | 87±1.1^c^ | 77±1.5^b^ | 72±1.2^ab^ | 68±1.3^a^ | <0.001 |
| Maltase activity (U/mg protein) | 20.1±1.2^b^ | 13.7±1.4^ab^ | 9.7±1.7^a^ | 9.3±1.4^a^ | 0.002 |
| Alkaline phosphatase activity (U/mg protein) | 45.6±1.2^b^ | 41.6±1.8^ab^ | 39.9±1.3^ab^ | 36.9±1.5^a^ | 0.019 |
| Leucine aminopeptidase (U/mg protein) | 141±1.4^c^ | 119±3.3^b^ | 100±1.8^a^ | 91±2.1^a^ | <0.001 |
| Mucosal folds height | 8.2±0.18^c^ | 7.3±0.16^b^ | 6.6±0.15^ab^ | 6.2±0.22^a^ | <0.001 |
| Mucosal folds fusion | 1.9±0.18^a^ | 2.6±0.14^b^ | 3.0±0.13^b^ | 3.7±0.13^c^ | <0.001 |
| Lamina propria width | 1.6±0.14^a^ | 2.3±0.14^b^ | 2.8±0.19^bc^ | 3.3±0.19^c^ | <0.001 |
| Lamina propria cellular infiltration | 1.3±0.1^a^ | 2.2±0.13^b^ | 2.8±0.15^c^ | 3.4±0.18^d^ | <0.001 |
| Submucosa width | 1.4±0.14^a^ | 2.0±0.13^b^ | 2.0±0.13^b^ | 3.5±0.16^c^ | <0.001 |
| Submucosa cellular infiltration | 1.3±0.12^a^ | 1.9±0.14^b^ | 3.1±0.17^c^ | 3.8±0.21^d^ | <0.001 |
| Enterocyte vaculization | 8.0±0.18^b^ | 7.5±0.14^b^ | 7.0±0.16^a^ | 6.7±0.13^a^ | <0.001 |
| Enterocyte nucleus position | 1.9±0.10^a^ | 2.0±0.13^a^ | 3.0±0.16^b^ | 3.3±0.14^b^ | <0.001 |
| *il-1β* expression | 1.00±0.03^a^ | 1.86±0.16^a^ | 3.32±0.02^b^ | 5.02±0.51^c^ | <0.001 |
| *il-8* expression | 1.01±0.11^a^ | 1.27±0.07^a^ | 4.11±0.80^ab^ | 7.86±2.11^b^ | 0.009 |
| *tnf-α* expression | 1.01±0.09^a^ | 1.98±0.08^ab^ | 3.38±0.31^bc^ | 5.37±0.84^c^ | 0.001 |
| *tgf-β* expression | 1.01±0.07^a^ | 2.63±0.12^b^ | 2.28±0.29^ab^ | 4.26±0.47^c^ | <0.001 |
| Serum diamine oxidase activity | 10.6±1.3 | 9.5±1.1 | 9.1±1 | 9.4±0.5 | 0.748 |
| Serum D-lactate level | 11.5±0.7 | 9.3±0.4 | 10.9±1.2 | 8.7±1 | 0.155 |
| Malondialdehyde level (nmol/mg protein) | 0.28±0.02^a^ | 0.33±0.01^a^ | 0.36±0.02^a^ | 0.50±0.01^b^ | <0.001 |
| Superoxide Dismutase activity (U/mg protein) | 44.4±2.3^b^ | 43.5±2.1^b^ | 42.2±1.9^b^ | 30.5±1.9^a^ | 0.005 |
| Calalase activity (U/mg protein) | 4.05±0.13^b^ | 3.40±0.20^ab^ | 2.88±0.06^a^ | 2.77±0.21^a^ | 0.002 |
| Glutathione level (mg/g protein) | 15.4±1^b^ | 11.1±0.8^a^ | 10.8±0.9^a^ | 9.2±0.7^a^ | 0.005 |
| Glutathione peroxidase activity (U/mg protein) | 26.7±1.3^c^ | 16.6±0.9^b^ | 13.1±1.5^ab^ | 10.3±1.1^a^ | <0.001 |
| Glutathione reductase activity (U/g protein) | 4.6±0.47^b^ | 3.78±0.22^ab^ | 3.34±0.14^ab^ | 2.9±0.14^a^ | 0.014 |
| Lysozyme (µg/mg protein) | 0.048±0.004 | 0.048±0.007 | 0.053±0.004 | 0.054±0.002 | 0.638 |
| Acid phosphatase activity (U/g protein) | 115±2^b^ | 101±3^ab^ | 95±6^a^ | 106±2^ab^ | 0.027 |
| Complement 3 level (µg/mg protein) | 35.6±2.9^c^ | 30.0±3.7^bc^ | 17.7±1.6^a^ | 20.5±1.2^ab^ | 0.004 |
| Complement 4 level (µg/mg protein) | 19.2±1.5^b^ | 16.7±1.7^ab^ | 11.4±1.8^a^ | 12.3±0.4^a^ | 0.017 |
| IgM level (µg/mg protein) | 68.9±4.7^b^ | 61.5±4.8^ab^ | 47.0±3.9^a^ | 44.8±0.9^a^ | 0.007 |

Data were expressed as mean values with the standard errors. Data in the same row with different superscript letters are significantly different (*P*< 0.05).

Abbreviations: FM, a basal diet; CGM20, about 20% of the corn gluten meal inclusion level to replace 33% fish meal protein in basal diet; CGM30, about 30% of the corn gluten meal inclusion level to replace 50% fish meal protein in basal diet; CGM40, about 40% of the corn gluten meal inclusion level to replace 67% fish meal protein in basal diet. *Il-1β*, interleukin-1 beta; *Il-8*, interleukin 8; *Tnf-α*, tumor necrosis factor α; *Tgf-β*, transforming growth factor β.
